# Vagal Nerve Stimulation-Modulation of the Anti-Inflammatory Response and Clinical Outcome in Psoriatic Arthritis or Ankylosing Spondylitis

**DOI:** 10.1155/2021/9933532

**Published:** 2021-05-27

**Authors:** C. Brock, S. E. Rasmussen, A. M. Drewes, H. J. Møller, B. Brock, B. Deleuran, A. D. Farmer, M. Pfeiffer-Jensen

**Affiliations:** ^1^Mech-Sense, Department of Gastroenterology and Hepatology, Clinical Institute, Aalborg University Hospital, Aalborg, Denmark; ^2^Department of Rheumatology, Aarhus University Hospital, Aarhus, Denmark; ^3^Department of Clinical Biochemistry, Aarhus University Hospital, Denmark; ^4^Steno Diabetes Center Copenhagen, Region Hovedstaden, Gentofte, Denmark; ^5^Centre for Trauma and Neuroscience, Blizard Institute, Wingate Institute of Neurogastroenterology, Barts and the London School of Medicine & Dentistry, Queen Mary University of London, London, UK; ^6^Institute of Applied Clinical Sciences, University of Keele, Stoke on Trent, UK; ^7^Copenhagen Center for Arthritis Research (COPECARE), Center for Rheumatology and Spine Diseases, Rigshospitalet, Glostrup, Copenhagen, and Department of Clinical Medicine, University of Copenhagen, Denmark

## Abstract

**Objectives:**

The vagal nerve exerts an essential pathway in controlling the cholinergic anti-inflammatory reflex. Thus, the study is aimed at investigating the acute effect of a noninvasive transcutaneous vagus nerve stimulation on clinical disease activity and systemic levels of inflammation in patients with psoriatic arthritis or ankylosing spondylitis.

**Methods:**

Twenty patients with psoriatic arthritis (PsA) and 20 patients with ankylosing spondylitis (AS) were included and stimulated bilaterally with a handheld vagal nerve stimulator for 120 seconds 3 times a day for 5 consecutive days. All patients were in remission. Cardiac vagal tone, clinical scores, CRP, and cytokine levels were assessed.

**Results:**

In PsA and AS, decreased heart rate was observed, confirming compliance. Furthermore, in PsA, a clear reduction of clinical disease activity associated with a 20% reduction in CRP was shown. In AS, a reduction in interferon-*γ*, interleukin- (IL-) 8, and 10 was shown. No side effects were described.

**Conclusion:**

This open-label study provides support for an anti-inflammatory effect of transcutaneous vagus nerve stimulation in patients with psoriatic arthritis and ankylosing spondylitis. The modulated immune response and reduced disease activity and CRP-levels raise the fascinating possibility of using neuromodulation as an add-on to existing pharmacological treatments.

## 1. Introduction

Psoriatic arthritis (PsA) and ankylosing spondylitis (AS) are chronic autoimmune diseases characterized by peripheral and spinal joint inflammation. The global prevalence of PsA and AS is approximately 0.5% [1–3], and the chronic inflammation of peripheral and spinal joints in PsA and AS leads to various degrees of impaired functionality associated with increased risks of cardiovascular comorbidities and mortality [4–6] and substantial socioeconomic expenses [7]. Currently, PsA and AS are typically treated with nonsteroidal anti-inflammatory drugs (NSAIDs) and/or disease-modifying antirheumatic drugs (DMARDs) such as methotrexate (MTX) [8] and targeted biological therapies, i.e., tumour necrosis factor-alpha (TNF-*α*) inhibitors, interleukin- (IL-) 17, and IL-12/23 inhibitors [9–12]. Frequent blood monitoring of the disease activity and presence of opportunistic infections is needed, and whilst most patients respond to these expensive treatments, a proportion of patients do not [13–15].

Circulating proinflammatory cytokines, such as TNF-*α*, has an important role in the pathophysiology of these disorders [16]. In a seminal animal study, Borovikova et al. showed the existence of the cholinergic anti-inflammatory pathway (CAP). Serum levels of TNF-*α* were decreased in endotoxin-treated animals that received electrical stimulation of the vagus nerve (VN) in comparison to vagotomised animals [17]. The anti-inflammatory effect is exerted through multiple neuroimmune interactions primarily via vago-vagal and vago-splenic pathways [18, 19], often referred to as the cholinergic anti-inflammatory pathway/reflex [20]. Many immune-mediated inflammatory disorders are characterized by a relative paucity of vagal tone, and therefore, vagal nerve stimulation (VNS) has been proposed as a potential anti-inflammatory intervention [18]. Clinical reduction of serum levels of TNF-*α* and clinical disease activity scores in rheumatoid arthritis [21] and C-reactive protein (CRP) in Crohn's disease have been reported [22], in response to invasive VNS-devices, which necessitates operative implantation with potential postoperative complications [23]. Consequently, novel noninvasive transcutaneous VNS (t-VNS) devices are emerging in the field of bioelectronics [24]. In healthy participants, bilateral t-VNS of the cervical part of the VN for 90 seconds caused a significant increase in cardiac vagal tone (CVT), a validated biomarker of efferent vagal tone, and a reduction in TNF-*α* lasting for up to 24 hours [25]. In rheumatoid arthritis, t-VNS resulted in reductions in disease activity, CRP, interferon-*γ*, and interleukin-10 [26], but hitherto, such transcutaneous devices have not been explored in the treatment of PsA or AS. We hypothesized that t-VNS would increase resting CVT and reduce the level of systemic inflammation and disease activity in such patients. Hence, this study is aimed at investigating the acute and short-term effect of t-VNS on CVT and the short-term effect of t-VNS on the disease activity and systemic level of inflammation.

## 2. Materials and Methods

### 2.1. Ethical Considerations

All participants provided written informed consent. The study was approved by the Ethical Committee in “Region Midt,” Denmark (1-10-72-199-16), the Danish Data Protection Agency (1-16-02-442-16), and the European Databank for Medical Devices (CIV-16-03-015125). The study was conducted in accordance to Good Clinical Practice (CPMP/ICH/135/95) and in compliance with the Declaration of Helsinki and its revised editions.

### 2.2. Study Design

**Figure 1:**
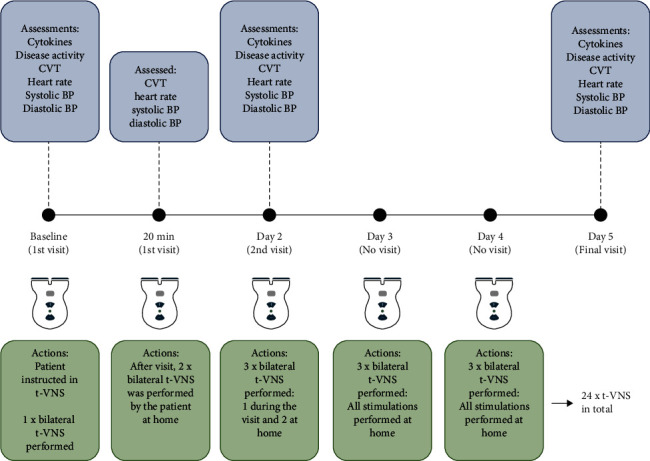
Schematic presentation of the study protocol.

This single-center, open-label, proof-of-concept study was designed to investigate the potential anti-inflammatory effects of t-VNS in two parallel cohorts diagnosed with PsA and AS. Participants were recruited from the Department of Rheumatology, Aarhus University Hospital, Denmark. Eligible participants, according to the inclusion and exclusion criteria, had their clinical disease activity measured using the DAS28-CRP and ASDAS scores, underwent noninvasive evaluation of autonomic parameters, and had venous blood drawn for the analysis of cytokines. Participants were then thoroughly instructed on how to deliver t-VNS, using the noninvasive, handheld stimulator (gammaCore; electroCore Inc. Basking Ridge, NJ, USA) to both the left and right cervical vagus nerves. In order to personalize stimulation to a therapeutic level, the t-VNS intensity was slowly increased until participants experienced a nonpainful mild pulling of the ipsilateral oral commissure. Participants then self-stimulated the left and right cervical vagus nerves using the gammaCore device three times daily (morning, afternoon, and evening) during the four-day intervention period (24 stimulations in total). Each stimulation lasted for 120 seconds. On the second study day, participants were asked to demonstrate the t-VNS to the study personnel in order to enhance patient safety, correct application, and study compliance. The study protocol is summarized in Figure 1. In addition to the baseline visit, study site visits took place at day 2 and day 5 including autonomic measures and venous blood sampling.

### 2.3. Study Participants

Eligible study participants included females and males with an established diagnosis of PsA or AS according to ACR criteria. Adult participants (>18 years) were included if they had no known contradictions to t-VNS such as known cardiovascular diseases including uncontrolled hyper/hypotension. Exclusion criteria included treatment with oral or intra-articular corticosteroids within the preceding five weeks or pregnancy (positive urine-HCG or lactating). None of the included patients had underwent previous vagotomy and/or had a currently implanted electrical or neurostimulating device.

### 2.4. Vagus Nerve Stimulation

Noninvasive t-VNS was performed using the handheld, portable gammaCore stimulator. The device contains two stainless steel electrodes, which deliver electrical stimulation to the cervical part of the vagus nerve. The electrical signal is comprised of small electrical bursts with a 1-millisecond duration (five 5 kHz sine waves, each lasting 200 milliseconds) repeated at 25 Hz. The low-voltage signal produced by the gammaCore can be varied according to tolerability but is limited to a peak voltage of 24 volts and a maximum output current of 60 mA when placed on the skin.

### 2.5. Cardiac-Derived Parameters

Autonomic measures were assessed by a portable ECG recording device (Faros 180°; Bittium, Oulu, Finland) connected to three ECG electrodes (Ambu BlueSensor P; Ambu, Copenhagen, DK). The electrodes were placed on clean and dry skin with the left and right arm electrodes positioned in the infraclavicular fossae and the left leg electrode near apex cordis. Five-minute resting recordings were performed on days 1, 2, and 5. The recordings were subsequently analysed using the bespoke software (ProCVT; ProBiometrics, London, UK) from which R-R intervals, HR, and cardiac vagal tone (CVT) were derived. CVT is a validated cardiometrically derived beat-to-beat measure of efferent vagal influence on the heart, and parasympathetic tone can be derived in recordings in epochs as short as 5 minutes, and details are described elsewhere [27]. CVT is measured on a linear vagal scale where 0 refers to full atropinisation. Any changes in heart rate (HR) of two consecutive heart beats at rest larger than 15 beats per minute (bpm) were removed from the recording as they were considered as artefacts due to sudden moves by the patient (e.g., coughing, sneezing, and change of position). Blood pressure (BP) was measured on the upper right arm using an electronic sphygmomanometer (UA-852; A&D Company Ltd., Tokyo, Japan).

### 2.6. Disease Activity

Disease activity was assessed on days 1, 2, and 5 via a physical examination and a combination of self-reported indices electronically entered in the Danish DANBIO database. Physical examinations included a count of tender and swollen joint(s) and an assessment of axial inflammation using the Bath Ankylosing Spondylitis Metric Index (BASMI) [28]. Calculated values of DAS28-CRP, BASMI, and Ankylosing Spondylitis Disease Activity Score (ASDAS) were then extracted from the DANBIO database. DAS28-CRP was originally developed to assess disease activity in rheumatoid arthritis, but performs well in clinical studies with patients with PsA [29–31]. Similarly, ASDAS was originally developed for patients with AS, but has shown to be valid in patients with peripheral spondyloarthropathies [32].

### 2.7. Cytokine Analysis

Venous blood was collected by experienced personnel at BY-lab, Aarhus University Hospital. Levels of C-reactive protein (CRP) were measured as part of a standard biochemical analysis, in addition to a variety of different biomarkers, at the hospitals' biochemical laboratory. Once collected, samples were centrifuged at 800 × g for 10 minutes, and the plasma was collected and frozen at -80°C. An electrochemiluminescence (ECL) immunoassay was then performed using two multiplex cytokine assays (V-PLEX Proinflammatory Panel 1 Human Kit and V-PLEX Custom Human Cytokine; Meso Scale Diagnostics, Rockville, Maryland, USA) and a plate reader (MESO QuickPlex SQ 120; Meso Scale Diagnostics, Rockville, Maryland, USA). The following cytokines were analysed: IFN-*γ*, IL-1*β*, IL-2, IL-4, IL-6, IL-8, IL-10, IL-12p70, IL-13, IL-17, IL-23, and TNF-*α*. Only measurements of IFN-*γ*, IL-8, IL-10, and TNF-*α* demonstrated data sets where <5% of the observations were below detection range.

### 2.8. Statistical Analyses

Data are presented as mean ± standard deviation (SD) or median and interquartile range (IQR) depending on data distribution as assessed by visual inspection of histograms and Q-Q plots. Group differences in demographics, clinical characteristics, and t-VNS aspects were assessed with Student's *t*-test, Mann-Whitney test, chi-squared, or Fisher's exact test. A mixed effect model and post hoc analyses were conducted on CVT, HR, BP, DAS28-CRP, and ASDAS and cytokines to test differences between pre- and posttreatment. CRP measurements below detection rates (<0.6) were assigned the value 0.60 for further analyses. Cytokine measurements below detection rate were assigned a value corresponding to “Limit of detection/√2” [33]. If >5% of data were missing due to too low values, the cytokines were not analysed and included in the study. Linear associations were assessed using Pearson's correlation. As this was a preliminary exploratory proof of principle study, a sample size calculation was not performed. *P* values <0.05 were considered statistically significant. All statistical analyses were performed using a standard software package (Stata Statistical Software, Release 14; StataCorp LLC, College Station, TX, USA).

## 3. Results

### 3.1. Participant Disposition, Demographics, and General Characteristics

**Figure 2:**
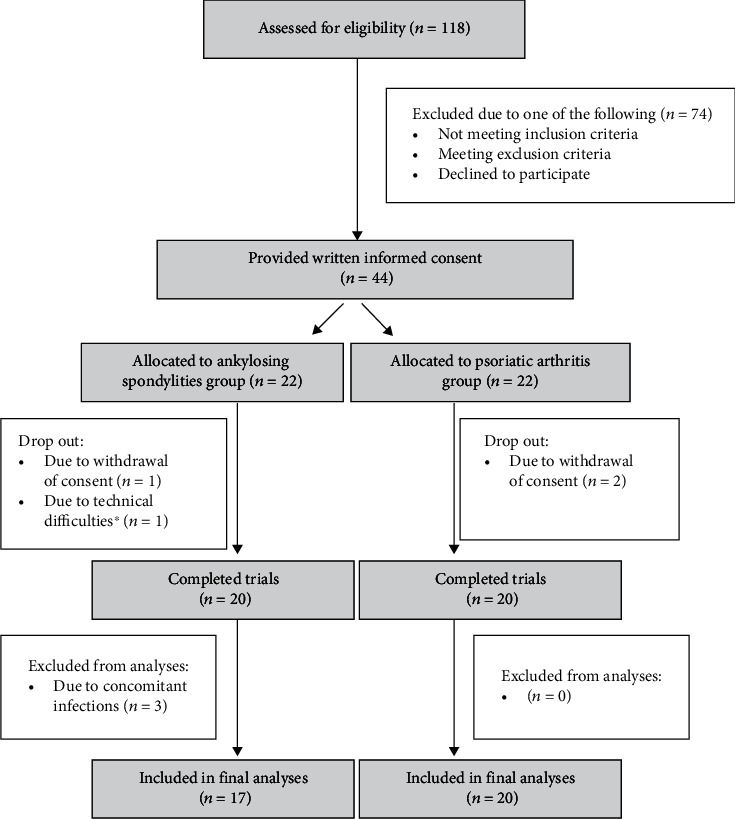
Flowchart illustrating screening process. ^∗^Temporary failure in the ECG-recording device.

A total of 118 possible participants were prescreened, and a total of 20 patients diagnosed with PsA and 20 patients diagnosed with AS were included in the study. For further details, see Figure 2. Due to concomitant infection (pneumonia and UTI) during the study, 3 patients diagnosed with AS were withdrawn by the study personnel, leaving 17 for final analyses.

The two cohorts differed in numbers treated with MTX usage, body mass index, and median stimulation amplitude. In other aspects, the groups displayed similar demographics; details are provided in Table 1.

### 3.2. Participants with Psoriatic Arthritis

*Cardiac-derived parameters* (*n* = 19): CVT recordings from one patient in the PsA group were uninterpretable due to significant movement artefact. The acute response to t-VNS was a significant decrease in HR 20 minutes after stimulation (71 bpm vs. 68, *P* = 0.019). Furthermore, t-VNS caused a significant reduction in median CVT from baseline to day 5 (5.73 LVS vs. 4.69, *P* = 0.017).

*Disease activity data* (*n* = 20): t-VNS reduced median ASDAS from baseline to day 5 (2.22 vs. 2.03, *P* = 0.012). The clinical composite score DAS28-CRP was unchanged, but in comparison to baseline t-VNS caused a reduction in median CRP on day 2 (3.23 vs. 2.72, *P* = 0.043) and day 5 (3.23 vs. 2.59, *P* = 0.001).

*Cytokine data* (*n* = 20): t-VNS induced an increase in TNF-*α* on the 5^th^ day (1.65 vs. 1.81, *P* = 0.005). Please see Figure 2 and Table 2 for details.

### 3.3. Participants with Ankylosing Spondylitis

*Cardiac-derived parameters (n =17)*: the acute response to t-VNS was a significant decrease in HR (69 bpm vs. 65, *P* = 0.028) and an increase in median CVT (5.38 LVS vs. 6.03, *P* = 0.027).

*Disease activity data* (*n* = 17): t-VNS did not change DAS28-CRP, ASDAS, or CRP.

*Cytokine data* (*n* = 17): t-VNS induced a decrease in IFN-*γ* (4.36 vs. 3.76, *P* = 0.02), IL-8 (3.83 vs. 3.03, *P* = 0.02), and IL-10 (0.46 vs. 0.42, *P* = 0.008) on the 2^nd^ day.

Please see Table 3 for details.

## 4. Discussion

This preliminary proof-of-concept report is the first to examine the potential anti-inflammatory effects of short-term t-VNS in patients with PsA and AS. t-VNS lowered HR in patients with PsA and reduced the objective biomarker CRP and clinical disease activity, despite slightly increased levels of the proinflammatory cytokine TNF-*α*. Taken together, we consider t-VNS as a promising add-on therapy to existing pharmaceutical intervention in PsA. In patients with AS, t-VNS lowered HR, increased the cardiac vagal tone, and reduced the proinflammatory cytokines IFN-*γ*, IL-8, and the anti-inflammatory cytokine IL-10, indicating modulation of the overall immune response in patients with AS.

### 4.1. The Link between Inflammation and Parasympathetic Tone

It has become increasingly accepted that the ANS—and the VN in particular—is involved in control and regulation of the immune system, the so-called neuroimmune interaction [34]. Tracey has described an inflammatory reflex in which biochemical signals of systemic inflammation are transmitted to the brain by afferent vagal nerve fibres [20]. In response to these, the vagal nerve exerts a combined anti-inflammatory effect by activation of the hypothalamic-pituitary-adrenal (HPA) axis, the cholinergic anti-inflammatory pathway/reflex, and the spleen. Taken together, although considerable uncertainty exists, the clinical effect that we observed has been suggested to be mediated by vagal modulation of nociplastic pain including the inhibition of inflammation, the sympathetic tone, and the pain neuromatrix—all of which are factors that contribute to development of central sensitization and chronic pain [35].

In addition, a growing body of evidence supports the notion that inflammation is associated with a sympathovagal imbalance. For example, in a murine model, Huang et al. demonstrated that lipopolysaccharide-induced endotoxemia caused a sympathetic-vagal disequilibrium with an overexcitation of the sympathetic nervous system [36]. These findings suggest that systemic inflammation may lead to the observed imbalance in the ANS, supporting the afferent mechanism. Moreover, long-term VN stimulation using an implanted VNS-device reduced disease activity and inhibited production of TNF-*α* in patients with rheumatoid arthritis [21], suggesting treatment efficacy of t-VNS in similar autoimmune and inflammatory diseases.

In healthy participants, there is a wide range of normal CVT, ranging from 2 to 18 LVS [37]. In the two patient cohorts with PsA and AS, we demonstrated that median CVT at baseline was in the lower end, but not outside the normal range. This contrasts the findings in patients with chronic pancreatitis, Crohn's disease, and type 1 diabetes, where chronic inflammation and neuropathy are considered to influence the autonomic dysfunction [38–40]. In AS patients, we demonstrated that in response to bilateral t-VNS, the CVT was increased and HR reduced, implicating increase parasympathetic tone. We did not assess CVT between the two stimulations, and thus, this data does not contain information on the t-VNS modulatory effect of stimulating right versus left cervical vagal nerve. Nevertheless, the findings resemble those observed in healthy participants [25] and support that t-VNS has a modulatory effect on the parasympathetic branch in these patients. This modulation did however not cause any alterations in clinical disease activity. In contrast, the PsA group failed to show acute increase in CVT but on the contrary demonstrated a decrease in CVT following stimulation. The different response in CVT, which is believed to reflect the efferent vagal tone accounting for 20% of the vagal nerve fibres, between the two cohorts may therefore be due to the differences in the pathogeneses of the diseases. For example, the promising effects of interleukin- (IL-) 17 and IL-23 inhibitors in patients with PsA (and not AS) suggest that there are differences in the nature of the inflammation between these patient groups [41]. Another explanation is that the response to t-VNS assessed with CVT seems to be influenced of systemic inflammation. For example, we have previously showed that CVT was raised in response to t-VNS in patients with rheumatoid arthritis in remission but not with flare [26], which may indicate that CVT is less robust in chronic inflammation; however, to clarify this, further investigations in other cohorts of inflammatory diseases are needed.

### 4.2. Vagal Nerve Stimulation and Anti-Inflammation

The basic scientific basis of the anti-inflammatory effect of t-VNS is widely accepted. Physiological modulation of the parasympathetic tone has also been demonstrated to have analgesic effects in healthy subjects. Using a validated acid-induced oesophageal pain model of central sensitization, it was shown that deep breathing, which increased parasympathetic tone, exerted an analgesic effect. This effect that was abolished by the coadministration of atropine suggests the cholinergic mediated signalling [42]. Moreover, t-VNS prevented the development of, and reversed established, acid-induced oesophageal hypersensitivity, by increasing parasympathetic tone [35]. Moreover, synergistically applied deep breathing and electrical stimulation of the auricular branch of the VN in healthy increased experimental thresholds of bone pain, indicating analgesic properties [43]. Furthermore, a single bilateral t-VNS caused a significant increase in CVT and a serum reduction in TNF-*α* lasting for up to 24 hours [25]. Taken together, these results suggest therapeutic implications for the management of pain. In addition, VNS has demonstrated anti-inflammatory effect in patients with Crohn's disease [22] and Koopman et al. demonstrated that long-term (84 days) invasive VNS significantly reduced disease activity and serum levels of TNF-*α* in patients with rheumatoid arthritis [21]. These intriguing results are in line with a novel study using t-VNS in 5 days, showing reduced disease activity (DAS28-CRP), CRP, and interferon-*γ* in patients with rheumatoid arthritis and flare [26].

In contrast to these studies, PsA patients in the current study were only stimulated during a 5-day protocol, and in response to that, we saw a clear reduction of clinical disease activity measured by the ASDAS score, associated with a 20% reduction in CRP, and such disease attenuation would not otherwise be expected in the normal natural history of this disorder. We saw that t-VNS modulated the immune response in both diseases.

The immune response is complex and can be considered as a dynamic balance between pro- and anti-inflammatory cytokines, as it requires and responds to continuous feedback mechanisms at the molecular, organ, and whole-host level. Interpretation of the observed decreased anti-inflammatory and increased proinflammatory cytokines should be done with caution because patients were investigated at relatively low levels of inflammation due to concomitant immunomodulatory treatment and disease remission *ab initio*; other studies have used lipopolysaccharide-stimulated blood samples, and the focus on the dynamic balance which may not necessarily be quantitative (e.g., equal concentrations of pro- and anti-inflammatory cytokines) but rather should be considered as a qualitative harmonization in downstream activation and inhibition [44].

## 5. Limitations

This study has some intrinsic limitations. Firstly, as this was an open-label study, it was conducted without blinding and thus the lack of a placebo-arm/sham stimulation limits any firm conclusion on the anti-inflammatory efficacy of t-VNS in PsA and AS. Secondly, the small sample size, although comparable to similar studies, limits the generalisability of these findings. Nevertheless, further work is now warranted in larger patient groups with higher disease activity, with a potential possibility of categorizing participants according to their current treatment regimens, e.g., biological and/or conventional DMARDs. Thirdly, the assessment of clinical disease activity may have been vulnerable to bias as the reduction in the clinical scores such as DAS28-CRP and ASDAS yields a subjective part, which involves an assessment of tender and swollen joints. However, the natural history of PsA and AS and the clear objective reduction in CRP levels in PsA support that an anti-inflammatory effect was achieved through t-VNS in the short five-day treatment period. Finally, in spite of our efforts in terms of education and training in t-VNS stimulation, we cannot guarantee that participants positioned the gammaCore correctly at every stimulation, and thus, insufficient stimulation may have occurred; however, the decreased HR supports that the t-VNS modulated the ANS in patients with PsA and AS.

In conclusion, this open-label preliminary report provides support for an anti-inflammatory effect of t-VNS in patients with PsA and AS, evident as convincing reduction in HR, disease activity, and CRP-levels. This raises the fascinating possibility of using neuromodulation as an add-on to existing pharmacological treatments; however, these initial findings warrant further investigation in larger randomized sham-controlled trials.

## Figures and Tables

**Table 1:** Patient characteristics.

	Psoriatic arthritis (*n* = 20)	Ankylosing spondylitis (*n* = 17)	*P* value
General characteristics^∗^			
Age, (years, median (IQR))	45 (34-54)	45 (38-51)	0.77
Female, no. (%)	11 (55%)	6 (35%)	0.33
Disease duration, (years, median (IQR))	7 (3-8.5)	4.5 (2.5-15.5)	0.78
Caucasian ethnicity, no. (%)	20 (100%)	17 (100%)	N/A
Current smoker, no. (%)	1 (5%)	1 (6%)	1.00
Daily caffeine use, no. (%)	18 (90%)	16 (94%)	1.00
Methotrexate, no. (%)	15 (75%)	1 (6%)	<0.0001
Methotrexate dose amongst users, (mg per week)	16.5 ± 1.4	25 ± 0	0.15
Prescription NSAID, no. (%)	13 (65%)	11 (65%)	0.99
NSAID prescription dose (mg per day, median (IQR))	1200 (1000-1800)	1000 (800-1200)	0.11
Height (cm)	176.0 ± 10.6	177.4 ± 8.3	0.66
Weight (kilogram)	86.3 ± 17.6	79.3 ± 16.3	0.22
Body mass index (BMI)	27.7 ± 3.9	25.1 ± 4.4	0.02^†^
Vagnus nerve stimulation^∗^			
Stimulations used (no./patient, median (IQR))	24 (24-25)	24 (22-24)	0.3
Stimulation amplitude (intensity) (intensity, median (IQR))	30 (29-35)	27 (25-30)	0.04^†^

^∗^Data are presented as mean ± SD unless otherwise indicated. ^†^Significant difference, *P* < 0.05.

**Table 2:** Result—psoriatic arthritis.

	Baseline	20 min	*P* value	Day 2	*P* value	Day 5	*P* value	Overall *P* value
Cardiometric data								
Cardiac vagal tone (LVS)^∗^	5.73 (0.72)	5.68 (0.72)	0.892	5.49 (0.72)	0.531	4.69 (0.71)	0.007	0.023
Heart rate (bpm)	71 ± 3	68 ± 3	0.017	69 ± 3	0.077	73 ± 3	0.307	0.002
Systolic BP (mmHg)	130 ± 4	130 ± 4	0.872	126 ± 4	0.150	133 ± 4	0.141	0.031
Diastolic BP (mmHg)	83 ± 3	85 ± 3	0.310	82 ± 3	0.710	85 ± 3	0.173	0.255
Disease activity								
DAS28-CRP^∗^	2.54 (0.16)	-	-	2.53(0.16)	0.876	2.45 (0.16)	0.162	0.309
ASDAS^∗^	2.22 (0.20)	-	-	2.07 (0.20)	0.063	2.03 (0.20)	0.012	0.033
CRP (mg/L)^∗^	3.23 (0.84)	-	-	2.72 (0.84)	0.043	2.59 (0.84)	0.001	0.004
Cytokines								
IFN-*γ* (pg/L)^∗^	4.96 (0.92)	-	-	5.19 (0.12)	0.735	6.27 (0.89)	0.101	0.089
IL-8 (pg/mL)	3.41 (0.30)	-	-	3.72 (0.30)	0.324	3.99 (0.30)	0.061	0.172
IL-10 (pg/mL)	0.29 (0.05)	-	-	0.26 (0.04)	0.281	0.30 (0.05)	0.482	0.193
TNF-*α* (pg/mL)	1.65 (0.17)	-	-	1.69 (0.17)	0.476	1.81 (0.17)	0.005	0.014

^∗^Data are presented as estimated mean and standard error.

**Table 3:** Results—ankylosing spondylitis.

	Baseline	20 min	*P* value	Day 2	*P* value	Day 5	*P* value	Overall *P* value
Cardiometric data								
Cardiac vagal tone (LVS)	5.59 (0.77)	6.56 (0.77)	0.014	5.75 (0.77)	0.692	5.22 (0.77)	0.361	0.008
Heart rate (bpm)	69 ± 3	65 ± 3	0.009	67 ± 3	0.401	68 ± 3	0.796	0.041
Systolic BP (mmHg)	131 ± 4	131 ± 4	0.822	129 ± 4	0.565	131 ± 4	0.920	0.861
Diastolic BP (mmHg)	83 ± 3	83 ± 3	0.900	83 ± 3	0.616	83 ± 3	0.900	0.911
Disease activity								
DAS28-CRP	1.78 (0.17)	-	-	1.76 (0.17)	0.598	1.77 (0.17)	0.845	0.868
ASDAS	1.75 (0.21)	-	-	1.66 (0.21)	0.128	1.67 (0.21)	0.139	0.220
CRP (mg/L)	3.02 (0.88)	-	-	3.08 (0.88)	0.784	3.09 (0.88)	0.211	0.408
Cytokines								
IFN-*γ* (pg/L)	4.36 (0.95)	-	-	3.76 (0.95)	0.017	4.44 (0.95)	0.440	0.055
IL-8 (pg/mL)	3.83 (0.33)	-	-	3.03 (0.35)	0.024	3.90 (0.33)	0.840	0.026
IL-10 (pg/mL)	0.46 (0.05)	-	-	0.42 (0.05)	0.008	0.44 (0.05)	0.090	0.024
TNF-*α* (pg/mL)	1.43 (0.19)	-	-	1.44 (0.19)	0.861	1.51 (0.19)	0.167	0.331

^∗^Data are presented as estimated mean and standard error.

## Data Availability

Data is available upon request to the corresponding author.
